# Treatment Effect and Safety of Nanoliposomal Irinotecan with Fluorouracil and Folinic Acid after Gemcitabine-Based Therapy in Patients with Advanced Pancreatic Cancer: A Multicenter, Prospective Observational Study

**DOI:** 10.3390/jcm11175084

**Published:** 2022-08-30

**Authors:** Masami Miki, Nao Fujimori, Keijiro Ueda, Lingaku Lee, Masatoshi Murakami, Yu Takamatsu, Yuzo Shimokawa, Yusuke Niina, Takamasa Oono, Terumasa Hisano, Masayuki Furukawa, Yoshihiro Ogawa

**Affiliations:** 1 Department of Hepato-Biliary-Pancreatology, National Hospital Organization, Kyushu Cancer Center, Fukuoka 811-1347, Japan; 2 Department of Medicine and Bioregulatory Science, Graduate School of Medical Sciences, Kyushu University, Fukuoka 812-8582, Japan; 3 Department of Gastroenterology, Kitakyushu Municipal Medical Center, Fukuoka 802-0077, Japan

**Keywords:** nanoliposomal irinotecan, advanced pancreatic cancer, second-line therapy

## Abstract

Although the combination of nanoliposomal irinotecan plus fluorouracil/folinic acid (nal-IRI/FF) exhibited survival benefits in gemcitabine-refractory patients with advanced pancreatic cancer (APC) in the phase III NAPOLI-1 trial, there is limited data on the efficacy and safety of this regimen in real-world settings in Japan. This multicenter, prospective observational study enrolled patients with APC who received nal-IRI/FF after a gemcitabine-based regimen from July 2020 to June 2021. We collected and analyzed clinical data and conducted survival and multivariate analyses. Thirty-one (78%) of the 40 patients had metastases. Nal-IRI/FF was the second-line therapy in 36 patients (90%). The median duration was 3.2 months. The disease control rate was 57%. The median progression-free survival and overall survival (OS) were 4.5 months (95% confidence interval [CI]: 2.8–5.5) and 7.4 months (95% CI: 5.1–10.6), respectively. Common ≥grade 3 toxicities included neutropenia (28%) and fatigue (23%). Fatigue led to treatment discontinuation in 6 out of 10 patients. Multivariate analysis showed that a neutrophil-to-lymphocyte ratio > 4 was a significant risk factor for a short OS (hazard ratio (HR) = 3.08, 95% CI: 1.21–7.85, *p* = 0.02). In conclusion, nal-IRI/FF is an appropriate treatment option for APC following gemcitabine-containing regimens.

## 1. Introduction

According to the 2018 Global Cancer Analysis, pancreatic cancer (PC) remains the fourth leading cause of cancer-related deaths. PC’s 5-year relative survival rate is 8%, which is the lowest among all types of cancer [1]. Approximately 80% of patients with PC are diagnosed at an unresectable, locally advanced, or metastatic disease stage [2] and have only one treatment option: chemotherapy.

Over the past two decades, gemcitabine (GEM) has been the standard first-line therapy for patients with advanced PC (APC). Subsequently, the FOLFIRINOX (FFX; a combination of oxaliplatin, folinic acid, irinotecan, and fluorouracil) [3] and GEM plus nab-paclitaxel (GnP) [4] demonstrated a considerable survival advantage in patients with metastatic PC (MPC) and have been accepted as the most common first-line therapies. However, there is an increasing demand for second-line therapies for those with unavoidable disease progression or drug intolerance. Nanoliposomal irinotecan (nal-IRI) is a new antitumor drug consisting of irinotecan sucralfate salt, a topoisomerase I inhibitor, encapsulated in liposome nanoparticles. Liposomal encapsulation facilitates a more efficient conversion of irinotecan to SN-38 (an active metabolite of irinotecan) than non-liposomal irinotecan [5]. This nal-IRI formulation contributes to drug stabilization by prolonging the circulation time and increasing the accumulation of SN-38 in the tumor [6,7].

In 2016, the global randomized phase III study NAPOLI-1 showed that the combination of nal-IRI and fluorouracil/folinic acid (nal-IRI/FF) resulted in significant survival benefits compared with nal-IRI alone or FF monotherapy in patients with MPC who previously received GEM-based therapy [8]. A randomized phase II trial targeting Japanese patients [9] also revealed that nal-IRI/FF provides a considerable survival benefit compared to FF alone.

Although some studies have reported the use of nal-IRI/FF in patients with APC, there are limited prospective studies that aim to record real-world data. In the present study, we conducted a multicenter prospective analysis to assess the therapeutic effects and safety of nal-IRI/FF in patients with APC after GEM-based therapy in Japan.

## 2. Materials and Methods

### 2.1. Patient Population and the Study Design

This study was a multi-institutional, prospective, observational analysis of patients with APC. Patients with unresectable PC who had previously received GEM-containing therapy and were administered nal-IRI/FF between July 2020 and June 2021 were enrolled. Patients who received regimens that included irinotecan, such as modified FFX, were excluded. Patients with a history of secondary malignancy in the previous five years were also excluded.

### 2.2. Treatment

As a standard regimen, the patients received an intravenous infusion of 70 mg/m^2^ nal-IRI over 90 min, followed by 2400 mg/m^2^ 5-FU and 200 mg/m^2^ levoleucovorin calcium as the initial doses every two weeks. Dose reduction was performed based on the toxicity (described in Section 2.3). We calculated the cumulative relative dose intensity (cRDI), which was defined as the average ratio (%) of the actual delivered nal-IRI and 5-FU to the planned amount throughout the treatment period, as in a previous study [10].

Therapy was discontinued if disease progression or unacceptable adverse events occurred. Continuation of the nal-IRI/FF treatment regimen after disease progression was allowed if the physician judged it feasible.

### 2.3. Evaluation of Efficacy and Toxicity

The primary endpoint was overall survival (OS), defined as the interval from the first administration of nal-IRI/FF until death or the occurrence of censoring. Secondary endpoints were progression-free survival (PFS), disease control rate (DCR), and safety. We performed serial computed tomography (CT) scans every 2.5 months or shorter, according to the Response Evaluation Criteria in Solid Tumors version 1.0 (RECIST, v1.0). The serum carbohydrate antigen 19-9 (CA19-9) response was defined as a decrease in the level of CA19-9 by more than 50% from baseline during nal-IRI/FF therapy. All surviving patients were censored at the last follow-up date, 28 February 2022.

All adverse effects were evaluated according to the National Cancer Institute Common Terminology Criteria for Adverse Events, version 4.0 (CTCAE, v4.0).

### 2.4. Ethical Standards

This human study was performed in accordance with the ethical standards of the 1964 Declaration of Helsinki and its later amendments. Informed consent was obtained from all patients, and the study was approved by the ethics committees of the Kyushu Cancer Center (approval number: 2020-30), Kitakyushu Municipal Medical Center (approval number: 202008006), and Kyushu University (approval number: 2020-290).

### 2.5. Statistics

Categorical variables are expressed as frequencies and proportions. Fisher’s exact test was used for comparison between the groups. A two-sided *p* < 0.05 was considered significant. Continuous variables are expressed as medians and interquartile ranges [IQRs], and values were compared using the Mann–Whitney U test. The Kaplan–Meier curve was drawn, and differences in survival were evaluated using the log-rank test. Univariate and multivariate analyses for survival time were performed using the Cox proportional hazard regression model. Significant variables with a *p*-value < 0.1 in the univariate analysis were included in the multivariate analysis. All statistical analyses were performed using the Easy R program version1.3.6 (Saitama Medical Center, Jichi Medical University, Saitama, Japan) [11].

## 3. Results

### 3.1. Patient Characteristics

Forty patients were enrolled in this study. Patient characteristics are shown in Table 1. The median age was 70.5 [62.5–72.0] years, and 19 patients (48%) were male. Nine patients had disease recurrence after curative surgical resection. The median disease-free survival of patients with surgical resection was 11.0 [9.2–30.2] months. The numbers of patients with an Eastern Cooperative Oncology Group (ECOG) Performance Status (PS) 0, 1, and 2 were 21 (52%), 18 (45%), and 1 (3%), respectively. Most of the patients (78%) had metastases. The numbers of patients with 1, 2, and ≥3 metastatic sites were 12 (30%), 14 (35%), and 5 (13%), respectively. One patient was homozygous for UGT1A1*6, and another was heterozygous for UGT1A1*28 and *6.

Thirty-six (90%) and four (10%) patients received nal-IRI/FF as second- and third-line therapies, respectively. Progressive disease and toxicity led to discontinuation of the previous therapy in 36 (90%) and four (10%) patients.

The median interval from the diagnosis with unresectable PC to the initiation of nal-IRI/FF was 6.6 [5.0–10.7] months. The median observation period was 7.4 [4.4–0.6] months.

### 3.2. Treatment Delivery and Dose Reduction or Discontinuation

The median treatment period was 3.2 [1.7–6.2] months, and the median number of cycles was 6 [3–10]. The administered doses and attributed reasons for dose reduction or discontinuation are summarized in Table 2. Twenty-eight (70%), nine (22%), and three (8%) patients received 100%, 80%, and 70% of the initial nal-IRI dose, respectively. The median overall cRDI score was 69.0% [54.5–86.5%].

### 3.3. Treatment Response

The treatment responses are summarized in Table 3. Among the evaluable 35 patients, partial response (PR) was observed in three patients (9%), stable disease (SD) in 17 patients (49%), and progressive disease (PD) in 15 patients (43%). The DCR was 57%. There were 8 CA19-9 responders (25%) among 32 patients with evaluable CA19-9 values.

### 3.4. Survival

**Figure 1:**
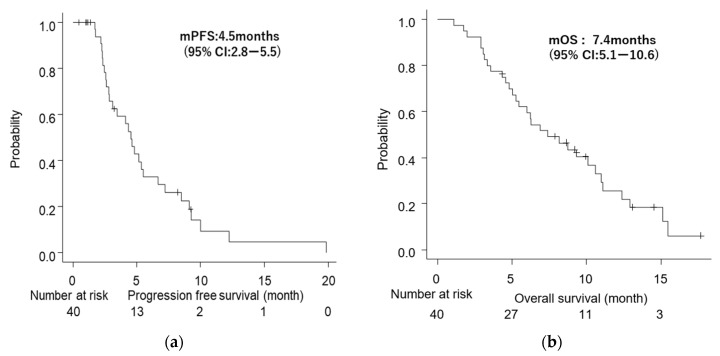
Kaplan–Meier survival analyses. (**a**) Progression-free survival (PFS) of all patients. (**b**) Overall survival (OS) of all patients; CI, confidence interval, and *p*-value were calculated using the log-rank test.

**Figure 2:**
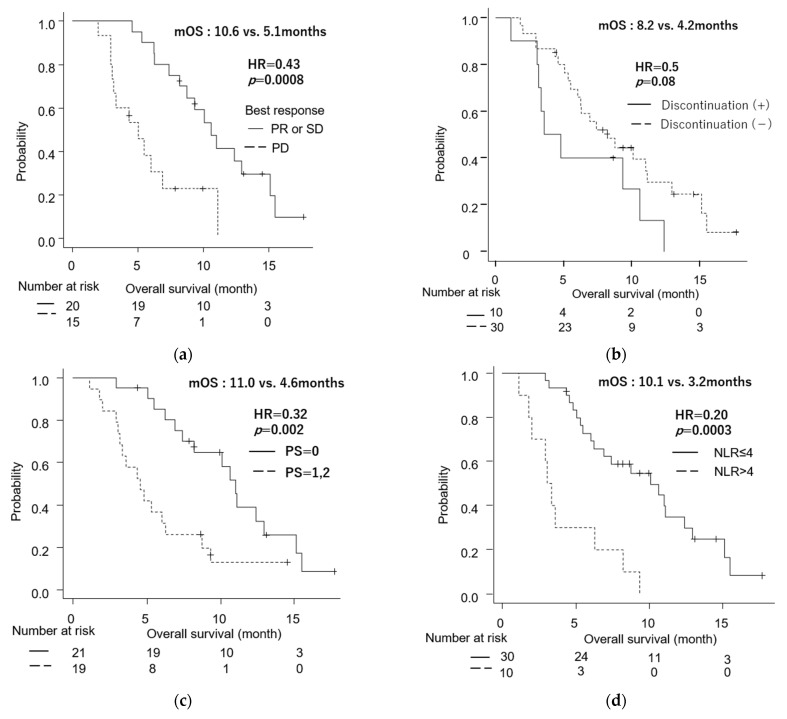
Kaplan–Meier survival analyses. (**a**) Overall survival (OS) with the best response = progressive disease (PD) versus stable disease (SD) or partial response (PR). (**b**) OS with treatment discontinuation versus without discontinuation. (**c**) OS with performance status (PS) = 0 versus PS = 1, 2. (**d**) OS with neutrophil-lymphocyte ratio (NLR) > 4 versus NLR ≤ 4. mOS, median overall survival; HR, hazard ratio. *p*-value was calculated using the log-rank test.

Median PFS (mPFS) was 4.5 months (95% CI: 2.8–5.5), and median OS (mOS) was 7.4 months (95% CI: 5.1–10.6) (Figure 1a,b). The mOS from the diagnosis with unresectable status was 16 months (95% CI: 13.2–19.5). There was no difference in OS between patients with metastasis and locally advanced disease (6.3 vs. 10.6 months, *p* = 0.51) (Figure S1). Regarding the relationship between treatment response and survival, the OS of patients with PD was significantly shorter than that of patients with SD and PR (5.1 vs. 10.6 months), with an HR of 0.43 (95% CI: 0.20–0.94; *p* = 0.0008) (Figure 2a). On the other hand, there was no significant difference in OS between CA19-9 responders and non-responders (11.1 vs. 7.4 months, *p* = 0.42).

### 3.5. Safety

Table 4 shows the adverse events (AE) during nal-IRI/FF treatment. The most common AEs included neutropenia (63%), general fatigue (63%), and leukopenia (60%). Eleven patients had grade 3 neutropenia. None of the patients experienced febrile neutropenia or ≥grade 3 thrombocytopenia. Regarding the severe (≥grade3) non-hematologic AEs, general fatigue was observed in nine patients, anorexia in two patients, and diarrhea, which led to acute renal dysfunction in one patient. Dose reduction was conducted due to neutropenia in nine (23%), general fatigue in nine (23%), and diarrhea in two (5%) patients. Ten patients (25%) required discontinuation of treatment. Six out of ten patients stopped the therapy due to general fatigue, and the treatment for the remaining was discontinued due to diarrhea, hemobilia, septic shock, and ileus. Six of the patients were moved to another line of chemotherapy.

### 3.6. Comparison between Patients Who Continued and Discontinued the Treatment Regimen

Although not significant, patients who discontinued therapy due to AEs (n = 10) tended to have worse survival than patients who continued treatment until disease progression (mOS: 8.2 vs. 4.2 months, HR = 2.0; 95% CI: 0.90–4.5, *p* = 0.08) (Figure 2b).

We compared the clinical characteristics of patients who discontinued nal-IRI/FF due to AEs with those of the patients who did not (Table S1). The rate of patients with ECOG PS ≥ 1 was significantly higher in those who discontinued the treatment than in those who did not. Furthermore, the interval from the diagnosis to initiation of nal-IRI/FF tended to be longer in patients who discontinued the treatment than in those who did not (median interval: 9.9 vs. 6.5 months, *p* = 0.08).

### 3.7. Univariate and Multivariate Analyses

We performed univariate and multivariate analyses to identify the possible predictive factors of PFS (Table S2) and OS (Table 5). There were no significant prognostic factors for PFS. Meanwhile, PS ≥ 1 (hazard ratio [HR]: 3.1, 95% CI: 1.4–6.6, *p* = 0.004), neutrophil-lymphocyte ratio (NLR) > 4 (HR: 4.9, 95% CI: 2.1–11.3, *p* = 0.0002), and body weight loss > 5% from diagnosis (HR: 2.3, 95% CI: 1.1–4.9, *p* = 0.03) were associated with worse OS in the univariate analysis. The Kaplan–Meier curve (Figure 2c) showed that the mOS in patients with ECOG PS ≥ 1 (4.6 months) at the start of nal-IRI/FF treatment was significantly shorter than that in patients with PS = 0 (11.0 months). Metastatic disease was not a poor prognostic factor. Multivariate analysis showed that NLR > 4 (HR = 3.1, 95% CI: 1.2–7.9, *p* = 0.02) was an independent predictive factor for shorter survival. There was a significant difference in OS between patients with NLR > 4 and NLR ≤ 4 (*p* = 0.00003), as shown in Figure 2d.

## 4. Discussion

In line with previous studies [9,12], this multicenter prospective analysis confirmed the survival benefits and manageable safety profile of the naI-IRI/FF regimen in patients with APC who previously received a GEM-containing regimen in Japan.

Although there was no difference in the CA19-9 response, the PFS and OS observed in the current study were longer than in previous reports. There may be several reasons for this finding. First, the patient population included only a small number of patients with PS = 2 (only one), a high proportion (90%) who received nal-IRI/FF as a second-line therapy, and a relatively high proportion (45%) who received post-study anti-cancer treatment.

The subpopulation analysis of NAPOLI-1 [12] reported the baseline characteristics of the Asian subgroup vs. the overall population: the higher rate of patients with baseline Karnofsky PS ≥ 90 (65% vs. 59%), ≤1 previous line of metastatic therapy (74% vs. 66%), and post anti-cancer treatment (50% vs. 31%), which were similar to the findings in our study population. These contributed to better outcomes in Asian patients than in the overall study population. The outcome of this study also might be attributed to the patient characteristics.

Second, in a previous study on the efficacy of nal-IRI/FF [11], patients who experienced no cancer progression with irinotecan treatment showed improved survival compared to those with prior cancer progression with irinotecan (mOS: 7.7 vs. 3.9 months). Smith et al. [13] and Yu et al. [14] showed a similar result in patients with APC who had been treated with irinotecan. Thus, we excluded patients who had received prior irinotecan-containing therapy to assess the effect of nal-IRI/FF without being confounded by the history of irinotecan administration. This exclusion criterion might have contributed to the better PFS and OS in the current study (mPFS = 4.5, mOS = 7.2) compared with those reported in the NAPOLI-1 trial (mPFS = 2.3, mOS = 6.1) or other studies [15,16,17]. Third, the inclusion of locally advanced PC, which was distinct from the inclusion criteria of the NAPOLI-1 study, might have caused a better outcome. However, there was no significant difference between patients with or without metastasis. Finally, there is the possibility that a lower frequency of CT assessment and a relatively high rate of censored patients due to longer OS might have affected the longer PFS found in this study.

Contrary to the NAPOLI-1 study, wherein more than 10% of all patients died during the study, or within 30 days of the last dose, there were no deaths within 30 days of the last dose in the present study. Thus, based on this finding and the fact that ten patients (25%) in our study could continue therapy for more than half a year, the nal-IRI/FF regimen can generally be considered well-tolerated in Japanese clinical practice. Neutropenia and anorexia were common AEs of nal-IRI/FF, as reported in a previous study; there were no significant differences in incidence between this study and the NAPOLI-1 trial [8]. The incidence of diarrhea was lower than that of the overall intention-to-treat population in the NAPOLI-1 study, and the incidence was comparable to that of the Asian sub-population in the NAPOLI-1 study [12]. However, fatigue had a much higher incidence and was the leading cause of therapy discontinuation. Other AEs with fatal outcomes, such as hemobilia and ileus, could not be considered treatment-related AEs, as they were probably complications of cancer itself. These incidences of various complications during the study might be explained as a result of the second-line setting where most patients have advanced diseases.

Intriguingly, the prognosis in disease-controlled patients with the best response of SD or PR was significantly better than that in patients with progressive disease. The results indicate that the response to the nal-IRI/FF regimen in patients previously treated with a GEM-based regimen considerably impacts prognosis.

The survival curve indicated that the discontinuation of nal-IRI/FF due to AEs was possibly associated with shorter OS, although it was not significant in this small population. The PS of patients who discontinued treatment was lower than that of patients who continued the treatment. This finding may help anticipate the clinical benefits of this regimen in individual patients.

The present study showed no significant difference in the starting doses of nal-IRI between the patients who continued and discontinued the treatment. However, clinical studies that involved starting dose reductions revealed that these reductions were not significantly associated with PFS or OS [18]. Moreover, they showed that the PS at the start of nal-IRI/FF treatment was not a poor prognostic factor when administered via a sufficiently reduced dose. Thus, a pre-emptive dose reduction should be considered to prevent serious AEs, especially in patients with low PS, as recommended in a Taiwanese study [15].

PS was associated with a short OS, consistent with a meta-analysis reporting that the baseline PS is a potent prognostic factor of PC after progression while on first-line GEM-based regimens [19]. Additionally, our multivariate analysis revealed that a high NLR was an independent poor prognostic factor. A high NLR (at the start of first-line therapy) has been widely reported to be associated with poor OS in APC [20,21]. In addition, a high NLR was a negative prognostic factor in the survival analysis of the NAPOLI-1 trial [22], which indicates the utility of NLR as a prognostic factor in second-line and first-line therapies.

Meanwhile, other reported factors, such as older age (≥65 years) and the presence of liver metastasis [22], related to poor prognosis, were not associated with short OS in our study. The difference in patient age distribution and liver metastasis volume might have affected the results. Further studies with larger sample sizes are required to develop a consensus on the prognostic factors of patients with APC receiving nal-IRI/FF treatment.

This study had several limitations. First, the sample size was too small to reach a definitive conclusion. Second, there was heterogeneity in the treatment dose and schedule due to the observational study design, where modification of the nal-IRI/FF regimen was allowed based on the physician’s decision. Third, we could not compare the efficacy of nal-IRI/FF therapy with that of other regimens such as FFX or modified FFX due to the single-arm fashion of our study. Further comparative studies are required to decide on an optimal regimen for patients with GnP failure. However, our real-world data might help establish an appropriate direction for using nal-IRI/FF.

Nal-IRI/FF is an effective sequential treatment after GEM-based treatment in patients with APC. This therapy should be administered under the careful management of AEs, including those triggered by an advanced disease state. The NLR may be a prognostic indicator of the therapeutic effect of nal-IRI/FF.

## Figures and Tables

**Table 1:** Patient characteristics.

	n/Median	(%)/[IQR]
Age	70.5	[62.5–72.0]
Sex-Male	19	(47.5)
Body Mass Index	20.9	[14.3–26.5]
Primary site		
Head/Body/Tail	17/12/11 (43/30/27)	
Diameter of the primary tumor (mm)	32	[18–75]
Presence of history of biliary drainage	9	(23)
History of surgical resection	9	(23)
Distant metastases	31	(78)
Metastatic site		
Liver	16	(40)
Peritoneum	13	(3)
Lung	6	(15)
Number of metastatic sites		
1	12	(30)
2	14	(35)
≥3	5	(13)
ECOG PS		
0/1/2	21/18/1 (52/45/3)	
Presence of UGT1A1 polymorphism	2	(5)
Reason for the discontinuation of the previous therapy		
Progressive disease	36	(90)
Toxicity	4	(10)
Line of therapy where nal-IRI/FF was administered		
Second-line	36	(90)
Third-line	4	(10)
First-line regimen		
Gemcitabine + nab-paclitaxel	39	(98)
Gemcitabine + S-1	1	(3)
Second-line regimen *		
S-1	3	(8)
Gemcitabine + nab-paclitaxel	1	(3)
The duration between the diagnosis and initiation of nal-IRI/FF (months)		
	6.9	[5.0–10.7]
Post-study anticancer therapy		
modified FOLFIRINOX	7	(18)
Gemcitabine + nab-paclitaxel	3	(8)
Oxaliplatin + fluorouracil/folinic acid	7	(18)
S-1	1	(3)
Observation period (months)	7.3	[4.4–10.6]

ECOG PS, Eastern Cooperative Oncology Group Performance Status; nal-IRI/FF, irinotecan plus fluorouracil/folinic acid. * Second-line regimen, regimen administered to patients who received nal-IRI/FF as third-line therapy.

**Table 2:** Administered dose and attributed reasons for dose reduction or discontinuation.

	Median	(%)/[IQR]
The initial dose of nal-IRI		
100%	28	(70)
90%	0	(0)
80%	9	(21.5)
70%	3	(7.5)
Duration of treatment (months)	3.2	[1.7–6.2]
Cycles of treatment (n)	6	[3–10]
Relative dose intensity (%)	69.0	[54.5–86.5]
Dose reduction during all cycles	20	(50)
Dose reduction due to AE in the first 4 cycles	14	(35)
Discontinuation due to AE	10	(40)

**Table 3:** Overall response to treatment with irinotecan plus fluorouracil/folinic acid (nal-IRI/FF).

Tumor Response ^§^	n	(%)
PR	3	(9)
SD	17	(49)
PD	15	(43)
DCR	20	(57)
**CA19-9 response**	n	(%)
responder	8	(25)
non-responder	24	(75)

PR, partial response; SD, stable disease; PD, progressive disease; NE, not evaluable; DCR, disease control rate. CA19-9, carbohydrate antigen 19-9. Responders: patients with a reduction rate of CA19-9 > 50%; non-responders: patients without a reduction rate of CA19-9 > 50%. ^§^ Five patients were not evaluable due to treatment discontinuation before the follow-up CT. Eight patients were not evaluable.

**Table 4:** Adverse events during irinotecan plus fluorouracil/folinic acid (nal-IRI/FF) treatment.

	All Grade		Grade 3/4		Required Dose Reduction		Required Discontinuation	
	No.	(%)	No.	(%)				
Hematologic								
Neutropenia	25	(63)	11	(28)	9	(23)		
Leukocytopenia	24	(60)	5	(13)				
Thrombocytopenia	11	(28)	0	(0)				
Anemia	14	(35)	2	(5)				
Non-hematologic								
General fatigue	25	(63)	9	(23)	9	(23)	6	(15)
Anorexia	16	(40)	2	(5)				
Diarrhea	11	(28)	1	(3)	2	(5)	1	(3)
Hypokalemia	2	(5)	0	(0)				
Dysgeusia	2	(5)	0	(0)				
Hemobilia			1	(3)			1	(3)
Septic shock			1	(3)			1	(3)
Ileus			1	(3)			1	(3)
Infusion reaction	1	(3)	0	(0)				

**Table 5:** Univariate and multivariate analyses of clinical factors at the start of irinotecan plus fluorouracil/folinic acid (nal-IRI/FF) treatment for predicting overall survival.

Variables at the Start of nal-IRI/FF Treatment	n	Univariate			Multivariate		
		HR	(95% CI)	*p*-Value	HR	(95% CI)	*p*-Value
Age							
≥70 y.o.	20	1.17	(0.57–2.38)	0.67			
<70 y.o.	20						
Sex							
male	19	1.06	(0.52–2.17)	0.86			
female	21						
ECOG PS							
=1 or 2	19	3.08	(1.43–6.63)	0.004	2.24	0.86–5.73	0.09
=0	21						
Stage							
metastatic	31	1.40	(0.57–3.46)	0.51			
locally advanced	9						
Liver metastasis							
present	19	1.12	(0.54–2.32)	0.67			
absent	21						
Peritoneal metastasis							
present	14	1.12	(0.54–2.33)	0.76			
absent	26						
Carcinomatosis *							
present	19	1.44	(0.71–2.93)	0.31			
absent	21						
NLR							
>4	10	4.88	(2.10–11.3)	0.0002	3.08	1.21–7.85	0.02
≤4	30						
CA19-9							
>1000 U/dL	20	1.38	(0.67–2.80)	0.37			
≤1000 U/dL	16						
GPS							
=2	11	2.08	(0.94–4.59)	0.07			
=0.1	29						
LMR							
<3	27	1.18	(0.55–2.52)	0.67			
≥3	13						
PLR							
>150	32	0.80	(0.35–1.83)	0.60			
≤150	8						
PNI							
<45	30	0.78	(0.35–1.71)	0.53			
≥45	10						
Bodyweight decrease from the diagnosis as an unresectable disease							
>5%	14	2.29	(1.09–4.85)	0.03	1.46	0.64–3.32	0.37
≤5%	23						
The interval from the diagnosis to the administration of nal-IRI/FF							
>6.6months	20	1.04	0.51–2.14	0.95			
≤6.6months	20						

* Carcinomatosis, presence of multi-metastatic sites; GPS, Glasgow prognostic score. NLR, neutrophil-lymphocyte ratio; PLR, platelet-lymphocyte ratio; LMR, lymphocyte-monocyte ratio; PNI, prognostic nutritional index.

## Data Availability

The data presented in this study are available on request from the corresponding author.

## Supplementary Materials

The following supporting information can be downloaded at: https://www.mdpi.com/article/10.3390/jcm11175084/s1: Figure S1: Kaplan–Meier survival analysis. Overall survival (OS) with metastatic pancreatic cancer versus locally advanced pancreatic cancer; Table S1: Comparison of clinical factors at the start of irinotecan plus fluorouracil/folinic acid (nal-IRI/FF) treatment between patients who continued and discontinued the treatment due to adverse events; Table S2: Univariate analysis of clinical factors at the start of irinotecan plus fluorouracil/folinic acid (nal-IRI/FF) treatment for predicting progression-free survival.
