# Supplementary material for: Treatment Effect and Safety of Nanoliposomal Irinotecan with Fluorouracil and Folinic Acid after Gemcitabine-Based Therapy in Patients with Advanced Pancreatic Cancer: A Multicenter, Prospective Observational Study
Source: J Clin Med. 2022 Aug 30;11(17):5084. doi: 10.3390/jcm11175084 (PMC9457338; doi:10.3390/jcm11175084)

Figure S1. Kaplan–Meier survival analysis. Overall survival (OS) with metastatic pancreatic cancer versus locally advanced pancreatic cancer

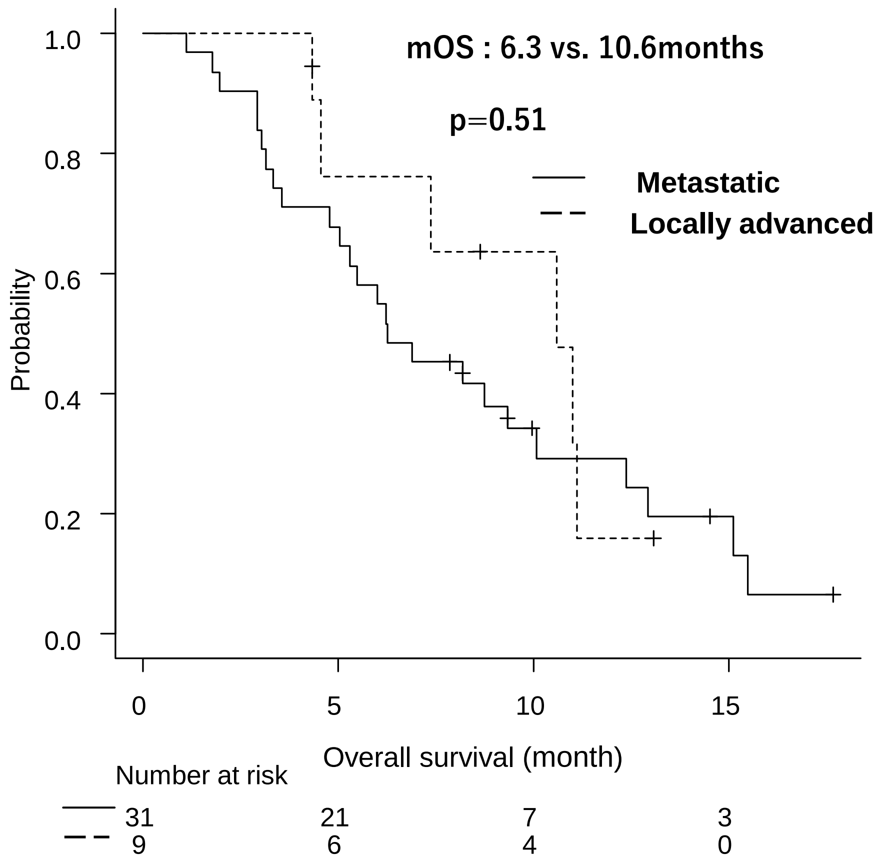

Supplement: Supplementary file 1 [file jcm-11-05084-s001.zip › Figure S1.pdf]
