# Supplementary material for: Treatment Effect and Safety of Nanoliposomal Irinotecan with Fluorouracil and Folinic Acid after Gemcitabine-Based Therapy in Patients with Advanced Pancreatic Cancer: A Multicenter, Prospective Observational Study
Source: J Clin Med. 2022 Aug 30;11(17):5084. doi: 10.3390/jcm11175084 (PMC9457338; doi:10.3390/jcm11175084)
Supplement: Supplementary file 1 [file jcm-11-05084-s001.zip › Supplementary tables.pdf]

Table S1: Comparison of clinical factors at the start of irinotecan plus fluorouracil/folinic acid (nal-IRI/FF) between the patients with and without discontinuation due to adverse event

	With discontinuation (n=10)				Without discontinuation (n=30)			
	n/median	(%) / [IQR]			n/median	(%) / [IQR]		p-value
Age	71.5	[69.5-72]			69.1	[60.8-72]		0.27
Sex- male	7	(70)			12	(40)		0.14
ECOG PS ≥1	9	(90)			10	(33)		**0.002***
Presence of liver metastases	4	(40)			12	(40)		1.00
Presence of carcinomatosis	5	(50)			14	(47)		0.85
The interval from the diagnosis* to initiation of nal-IRI	9.9	[3.2-24.8]			6.5	[2-20.3]		**0.08**
CA19-9 (U/mL)	873	[34-9594]			1200	[34-1100000]		0.97
GPS ≥2	5	(50)			6	(20)		**0.07**
Alb (g/dL)	3.70	[2.75-4.0]			3.55	[3.2-3.7]		0.79
CRP (mg/dL)	1.09	[0.35-2.73]			0.50	[0.05-1.36]		**0.08**
The initial dose of nal-IRI (%)	100	[75-100]			95	[73-100]		0.86
Dose reduction within the first four cycles	4	(40)			10	(33)		0.72

CRP, C-reactive protein; Carcinomatosis, presence of multi-metastatic sites; GPS: Glasgow prognostic score; * diagnosis, defined as diagnosis with unresectable status

Table S2: Univariate analysis of the clinical factors at the start of irinotecan plus fluorouracil/folinic acid (nal-IRI/FF) for progression-free survival

Variables	n		HR	(95% CI)	p-value
Age					
≥ 70 y.o.	20		1.20	5.56-2.57	0.65
< 70 y.o.	20				
Sex					
male	19		0.82	0.38-1.77	0.62
female	21				
ECOG PS					
=1 or 2	19		1.34	0.61-2.94	0.61
=0	21				
Presence of liver metastasis					
present	19		1.19	0.53-2.64	0.67
absent	21				
Presence of peritoneal metastasis					
present	14		1.97	0.83-4.69	0.12
absent	26				
Carcinomatosis					
present	19		0.82	0.39-1.76	0.62
absent	21				
NLR					
> 4	10		1.66	0.56-4.90	0.36
≤ 4	30				
CA19-9					
>1000 U/dL	20		0.82	0.38-1.76	0.61
≤ 1000 U/dL	16				
GPS					
=2	11		1.58	0.58-4.25	0.36
=0,1	29				
LMR					
< 3	27		0.70	0.32-1.59	0.37
≥ 3	13				
PLR					
>150	32		1.67	0.57-4.88	0.35

≤150	8				
PNI					
< 45			0.57	0.24-1.31	0.19
≥45					
BW decrease compared with diagnosis					
>5%	14		1.78	0.74-4.29	0.20
≤5%	23				
The interval from the diagnosis* to the administration of nal-IRI					
> 6.6months	20		0.74	0.36-1.51	0.40
≤ 6.6months	20				

Carcinomatosis, presence of multi-metastatic sites; GPS: Glasgow prognostic score

NLR, neutrophil-lymphocyte ratio; PLR, platelet-lymphocyte ratio; LMR, lymphocyte-monocyte ratio;

PNI, prognostic-nutritional index;
